# Sex differences in youth elite swimming

**DOI:** 10.1371/journal.pone.0225724

**Published:** 2019-11-22

**Authors:** Jonathon W. Senefeld, Andrew J. Clayburn, Sarah E. Baker, Rickey E. Carter, Patrick W. Johnson, Michael J. Joyner

**Affiliations:** 1 Department of Anesthesiology and Perioperative Medicine, Mayo Clinic, Rochester, Minnesota, United States of America; 2 Department of Health Sciences Research, Mayo Clinic, Jacksonville, Florida, United States of America; Universidade Federal de Mato Grosso do Sul, BRAZIL

## Abstract

**Background:**

The timing and magnitude of sex differences in athletic performance during early human development, prior to adulthood, is unknown.

**Objective:**

To compare swimming velocity of boys and girls for all Olympic-length freestyle swimming events to determine the age of divergence in swimming performance.

**Methods:**

We collected the all-time top 100 U.S. freestyle swimming performance times of boys and girls age 5 to 18 years for the 50m to 1500m events.

**Results:**

Swimming performance improved with increasing age for boys and girls (*p*<0.001) until reaching a plateau, which initiated at a younger age for girls (15 years) than boys (17 years; sex×age; *p*<0.001). Prior to age 10, the top 5 swimming records for girls were 3% faster than the top boys (*p*<0.001). For the 10^th^-50^th^ places, however, there were no sex-related differences in swimming performance prior to age 10 (*p* = 0.227). For both the top 5 and 10^th^-50^th^ places, the sex difference in performance increased from age 10 (top 5, 2.5%; 10^th^-50^th^ places, 1.0%) until age 17 (top 5, 7.6%; 10^th^-50^th^ places, 8.0%). For all places, the sex difference in performance at age 18 was larger for sprint events (9.6%; 50-200m) than endurance events (7.1%; 400-1500m; *p*<0.001). Additionally, the sex-related difference in performance increased across age and US ranking from 2.4% for 1^st^ place to 4.3% for 100^th^ place (*p*<0.001), indicating less depth of performance in girls than boys. However, annual participation was ~20% higher in girls than boys for all ages (*p*<0.001).

**Conclusion:**

The top 5 girls demonstrated faster swimming velocities and the 10^th^-50^th^ place girls demonstrated similar swimming velocities than boys (until ~10 years). After age 10, however, boys demonstrated increasingly faster swimming velocities than girls until 17 years. Collectively, these data suggest girls are faster, or at least not slower, than boys prior to the performance-enhancing effects of puberty.

## Introduction

Recent high profile cases have raised controversy about whether transgender athletes and XY (intersex) women with differences in sexual development should be allowed to participate in competitions restricted to women, for example Caster v. IAAF [1]. Stemming from the recognized performance-enhancing effects of androgens [2–6], regulation of endogenous androgen levels is now required to be eligible for participation in many women’s sports competitions. To better understand the timing and magnitude of sex differences in athletic performance during human development we examined elite swimming performances in youth as a proxy to estimate the expected divergence of athletic performance between girls and boys during periods of low androgen concentrations (pre-puberty) and increasing concentrations of androgens throughout puberty. As reviewed previously [6], human androgens—primarily testosterone and its related metabolite dihydrotestosterone (DHT)—are key factors in the development of muscle, bone and hemoglobin. Androgens largely contribute to bigger and more powerful muscle mass, higher hemoglobin concentrations (and subsequent oxygen carrying capacity), and greater bone strength in men than women [6]. In combination, these androgen-driven and sex-based differences in muscle, bone and hemoglobin contribute to a ~10% higher maximal oxygen consumption capacity (${\dot{\text{V}}\text{O}}_{2\text{max}}$) in men compared with women [7, 8].

Androgen levels are not different between the sexes prior to puberty, however, after completion of puberty, circulating testosterone levels are on average ~10–20 times greater in men than children or women at any age [9, 10]. This sex-based difference in circulating testosterone is the basic premise to explain why men have faster performance times than women in many time-based sports including running, cycling, swimming, rowing, etc. [11–17]. Thus, prior to puberty, it would be theorized that sex differences in performance between boys and girls would be negligible—which has been observed previously in athletes ~10–12 years of age [5, 18]. However, the sex-based differences in performance prior to age 10 are unknown and there is no previous data on long distance swimming which likely has the smallest influence from sociocultural biases [13]. Historically, women have had less opportunity to participate in most sports than men, and these differences in opportunity are suspected to contribute to the larger sex differences in performance than would be predicted based on physiological differences between the men and women [14, 19]. Women have been permitted to participate in swimming at the highest levels for many years (since ~1912) and currently more girls typically participate than boys, thus, swimming is an ideal ‘experiment of nature’ for this question [15]. Elite swimmers are also generally homogenous for high socio-economic status, meaning that sex differences in nutrition or access to medical care are unlikely [20]. There is intensive training from a young age, and practices and competitions are inclusive of both sexes. Additionally, standardized environmental conditions along with state of the art facilities are widely available during championship competitions.

Accordingly, the objective of our study was to determine the age of the divergence of swimming performance between elite boys and girls. To our knowledge, our study is the first to analytically investigate the role of normal human hormonal changes on sex-related differences in sprint and endurance performance in elite youth swimming. We hypothesized that: 1) there would be no sex-differences in swimming performance of girls and boys with similar and low testosterone concentrations (pre-pubescent years), 2) boys would be faster than girls after the initiation of puberty, and 3) the faster performance of boys would plateau after age 16, as androgen concentrations plateau [21].

## Materials and methods

### Methods

Finishing times of the top 100 All-Time Freestyle Swimming Records for Long Course Meters for boys and girls between 5 and 18 years of age in one-year age brackets were analyzed for all distances with full datasets (*n* = 100). Swimming times were downloaded from the USA Swimming Database (https://www.usaswimming.org /Home/times/data-hub) for six freestyle swimming distances from 50 to 1500 meters (50, 100, 200, 400, 800 and 1500 m) on April 4, 2019. Average swimming velocity (m·min^-1^) was calculated from the finishing time as: (race distance) × (finishing time)^-1^. Sex differences in swimming velocity were calculated for each place and event distance as: [(boy’s velocity)–(girl’s velocity)] × (boy’s velocity)^-1^ × 100%. The reduction in swimming velocities of boys and girls across world record place (between 1^st^ and 100^th^ place) was calculated as: (velocity of *n*^th^ place) × (velocity of 1^st^ place)^-1^ × 100%, for *n* = 1 to 100. Participation data was accessed via publicly-available membership demographics reports prepared by the USA Swimming Member Services staff for 2015 to 2018 (https://www.usaswimming.org). Additionally, circulating testosterone concentrations of a nationally representative sample of the United States population were downloaded from the National Health and Nutrition Examination Survey (NHANES) coordinated and conducted by the Centers for Disease Control and Prevention (CDC) (https://wwwn.cdc.gov/nchs/nhanes/Search/DataPage.aspx?Compon ent = Laboratory). As described previously [22], testosterone was quantified via isotope dilution liquid chromatography tandem mass spectrometry (ID-LC-MS/MS) based on the National Institute for Standards and Technology’s reference method, optimized by the CDC. This analytical quantification method initiated in 2013–2014 testing cycle, and data were analyzed for two consecutive testing cycles (2013–2014 and 2015–2016). These data are representative of the national population in demographic characteristics, and notably, are not specific to an elite-athletic population. All procedures accessed public information and did not require ethical review as determined by the Mayo Clinic Institutional Review Board in accordance with the Code of Federal Regulations, 45 CFR 46.102, and the *Declaration of Helsinki*.

### Statistical analysis

Data were reported as means ± SD within the text. Separate full factorial univariate analyses of variance (ANOVAs) were used to compare the dependent variables (swimming velocity and relative performance (%1^st^ place) of boys and girls, and sex differences in swimming velocity) between three independent variables [age (5–18 years), US ranking (1^st^-100^th^) and event distance (50 m– 1500 m)]. *Post hoc* analyses (Tukey’s HSD multiple comparisons) were used to test for differences between pairs within a data set when significant main effects or interactions were identified for age, US ranking or event distance. Recognizing early puberty may exhibit high statistical leverage on observed sex effects; a sensitivity analysis was conducted by filtering the data to only consist of the top 10^th^ through 50^th^ performance times by age, sex and distance. *Post hoc* Student’s t-tests were used to test for differences between boys and girls when a significant interaction of sex was identified. Bonferroni corrected *p*-values for multiple comparisons (*p* < 0.025) were used for all *post hoc* analyses. Pearson correlation coefficients (r) were used to determine associations between the sex difference in swimming performance and average circulating testosterone concentrations. For all other analyses, significance was determined at *p* < 0.05. All analyses were performed with IBM Statistical Package for Social Sciences version 25 statistical package (IBM, Armonk, NY, USA) and R version 3.4.2 (Vienna, Austria).

## Results

**Fig 1:**
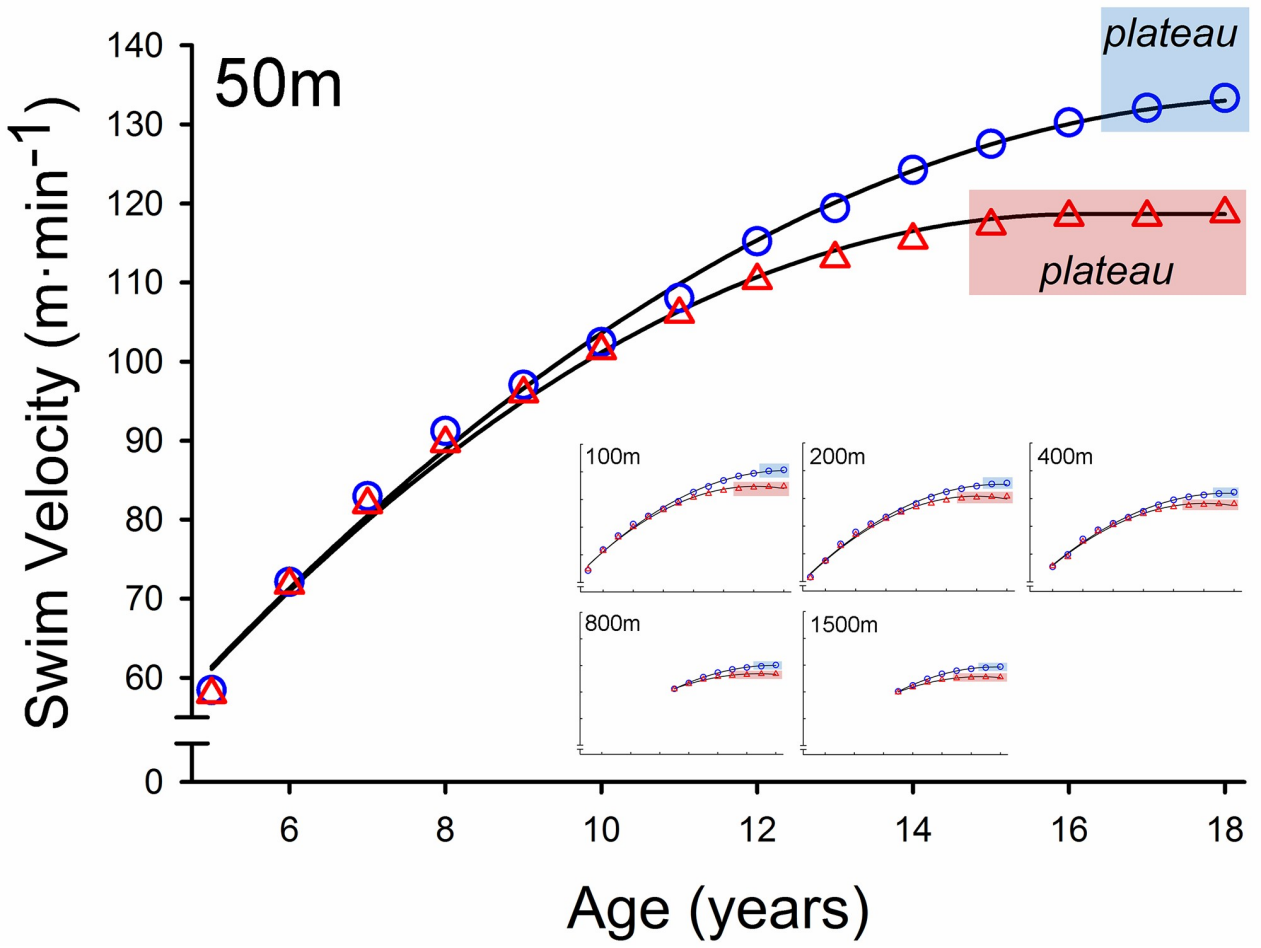
Elite swimming performance. Mean swimming velocity from 5 to 18 years of the top 100 fastest US boys (blue circles) and girls (red triangles) for the 50, 100, 200, 400, 800 and 1500m freestyle swimming distances.

For boys and girls, swimming velocity improved with advancing age according to a quadratic growth curve that was reproducible for each swimming distance (Fig 1). The quadratic growth curve demonstrates rapid improvements in swimming velocity up to 10 years of age after which the age-related improvement in performance slows and approaches a plateau (horizontal asymptote). There were many distinct differences between the age-related performance enhancement curves between girls and boys however. The plateau of swimming velocity was 8.4% lower for girls than boys (*p*<0.001), and the age at which the plateau in performance initiated was younger for girls (15 years) than boys (17 years) for all swimming distances aggregated (*p*<0.001). These data indicate that boys had faster swimming performances than girls particularly at older ages, thus, there was a sex-related difference in swimming performance that increased with age (*p*<0.001).

**Fig 2:**
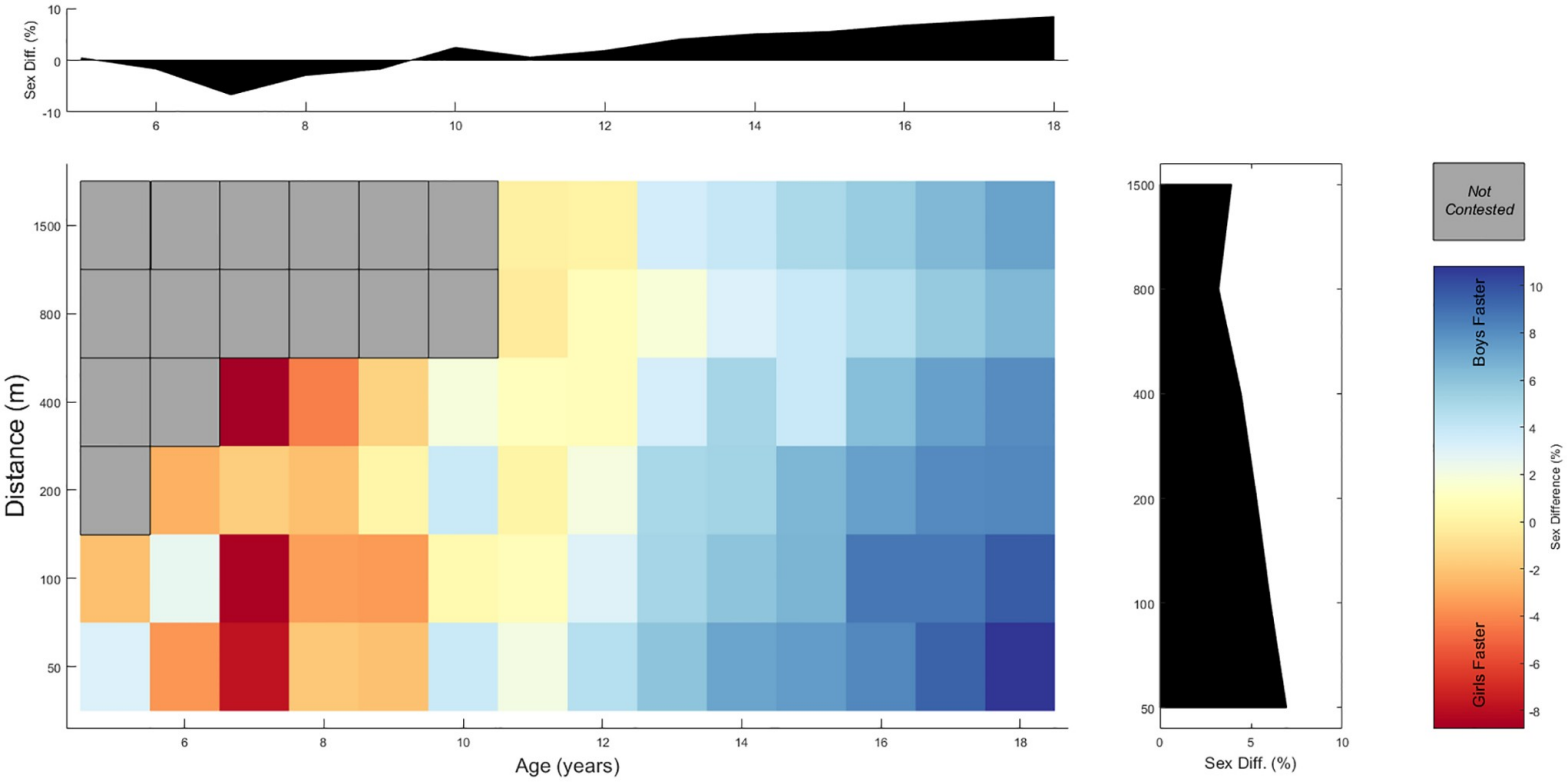
Sex differences in performance of the top 5 places. The primary plot (heat map) displays the sex differences in swimming velocity (% boy’s swimming velocity) of the *top 5 US rankings* in each freestyle event distance and age, negative values (red) indicate faster performance of girls. The top displays the mean sex difference across age, and the right plot displays the mean sex difference across swimming event distance.

**Fig 3:**
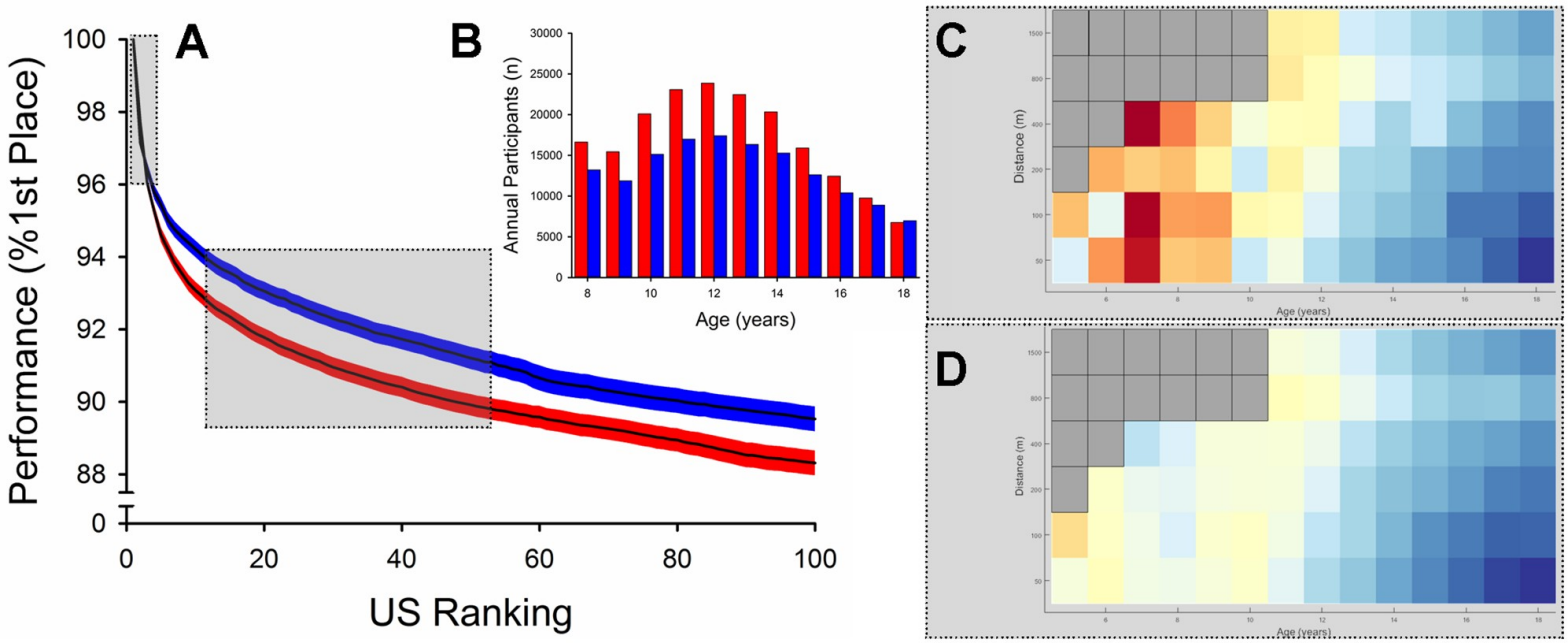
Relative performance decline across US ranking. The decline in swimming performance (% 1^st^ Place) across US ranking for boys (blue) and girls (red; mean ± 95% confidence interval; Panel A). The average annual membership numbers (Panel B) for boys (blue) and girls (red) of USA Swimming. The heat maps (Panels C and D) display the sex differences in swimming velocity of the top 5 US Rankings (Panel C) and the 10^th^-50^th^ US Rankings (Panel D) using the same color values displayed in Fig 2.

Considering the most elite competitors in the top 5 places, girls had 3% faster swimming performance than boys prior to age 10 (*p*<0.001; Fig 2). However, considering the 10^th^-50^th^ places, there were no sex-related differences in swimming performance between boys and girls prior to age 10 (*p*>0.05; Fig 3D). In the 50m for ages 5–9 years, for example, the average sex-related difference in performance was -2.5% for top 5 (indicating faster performance for girls) and 1.2% (indicating faster performance for boys) for the 10^th^-50^th^ places. Importantly, for both analyses, boys do not exhibit statistically faster swimming velocities than girls at young ages (<10 years). For both the top 5 and the 10^th^-50^th^ places, pairwise comparisons indicated that the sex difference in performance increased from age 10 (top 5, boys 2.5% faster; 10^th^-50^th^ places, boys 1.0% faster) incrementally increased for each age until the sex difference plateaued at age 17 (top 5, boys 7.6% faster; 10^th^-50^th^ places, boys 8.0% faster). Thus, beginning at age 10, boys had faster swimming performance than girls and the sex-difference in performance plateaued at age 17 (Fig 3C & 3D). For top 100 places aggregated, the sex-related difference in performance of 17–18 year olds was larger for the sprint distance events (9.6%; 50-200m) compared with the endurance distance events (7.1%; 400-1500m; *p*<0.001). The larger sex-related differences in performance for sprint distance events were observed for all ages (Fig 2).

Comparison of the relative reductions in velocity between the 1^st^ and 100^th^ place (age groups and event distances aggregated) demonstrated that the girls had greater reductions in relative velocity across place than boys (Fig 3A; sex × place, *p*<0.001). The average 100^th^ place US record holder swam at 90.7 ± 8.8% the velocity of the first place US record holder for the boys and 89.3 ± 9.0% for the girls (age groups and distances pooled). Thus, the sex difference in swimming performance progressively increased with US record place between first place (boys 2.4% faster) to 100^th^ place (boys 4.3% faster) across all ages and distances (*p*<0.001). These data indicate that there was less depth of performance in girls than boys (Fig 3). Despite the lesser depth of performance in girls, annual participation was higher in girls compared to boys (*p*<0.001; Fig 3B).

**Fig 4:**
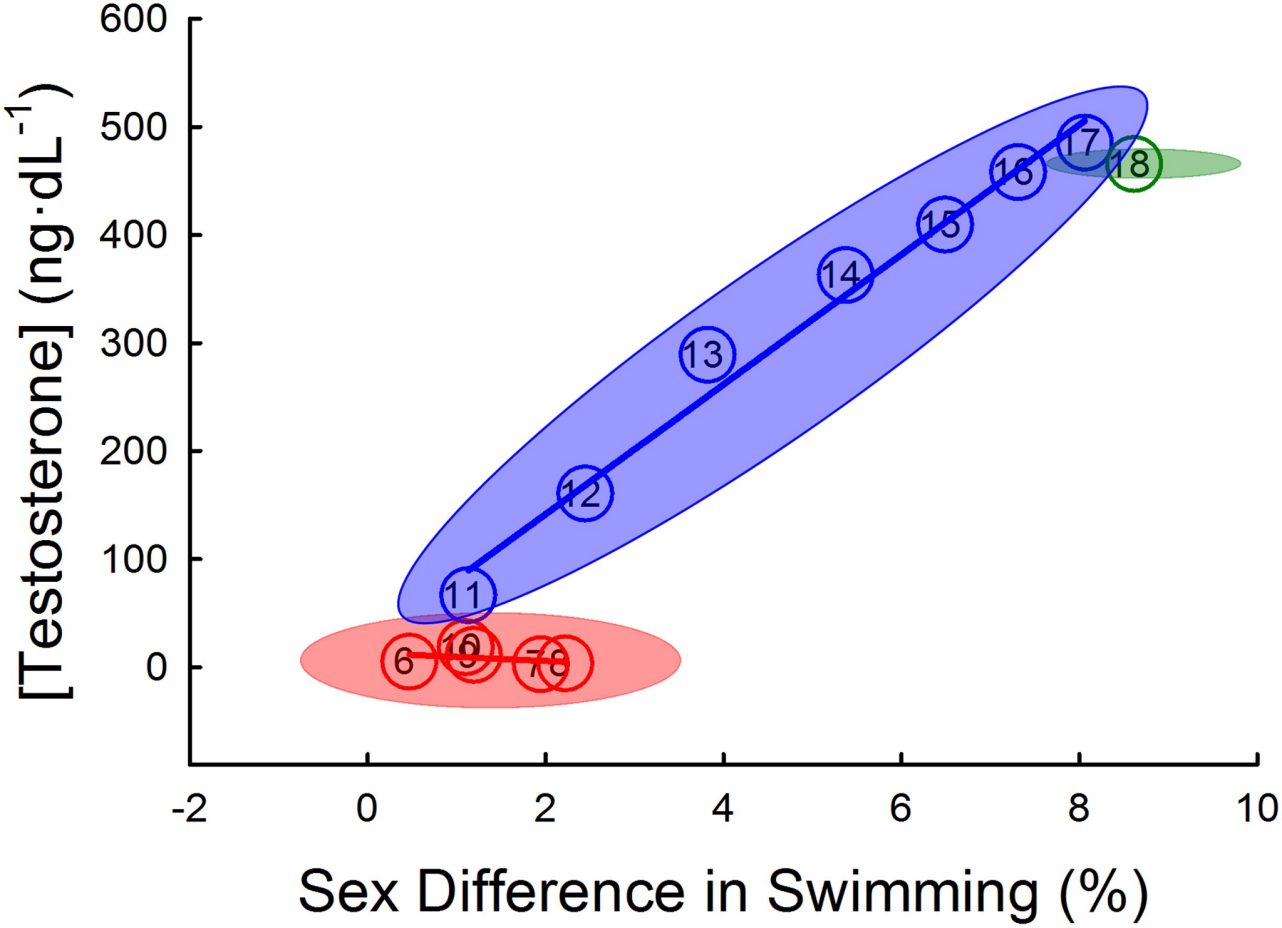
Correlation of boy’s serum testosterone and sex differences in swimming performance across age. The mean circulating testosterone concentrations from NHANES database were strongly, linearly correlated with the mean sex difference in swimming performance during the years of rapidly increasing testosterone levels for boys (11 to 17 years; *p*<0.001, *r* = 0.990), but not during the years of low testosterone for boys (6 to 10 years; *p* = 0.500). Each circle represents the mean sex difference in swimming for each age group pooled for all race distances and places (x-axis) and mean circulating testosterone level for boys (y-axis) with the age group denoted using corresponding Arabic numeral within each circle. The colored ellipses represent the standard error of three separate groupings for the correlation analysis, low-testosterone group (6–10 years, red), increasing testosterone group (11–17 years, blue) and plateaued testosterone group (18 years, green).

Circulating testosterone concentration data comprised results from 2,085 measurements. Boys had a greater than 100-fold increase in serum testosterone from ages 6 to 18 (3.6 ± 16.4 to 482.0 ± 232.0 ng∙dL^-1^, *p*<0.001), and this testosterone level began to plateau at 16 years. During the years of low testosterone for boys (<10 ng∙dL^-1^)—6 to 10 years—there was no association between testosterone (*p* = 0.500). However, during the years of rapidly increasing testosterone levels for boys—11 to 17 years—mean testosterone was strongly, linearly correlated with the mean sex difference in swimming performance (pooled for all race distances and places; *p*<0.001, *r* = 0.990). See Fig 4.

## Discussion

Using ‘big data’ as a proxy to estimate the ergogenic advantage of androgens in boys compared to girls, we determined the age of the sex-related divergence in elite swimming performance. As expected, boys had faster swimming performance than girls at 18 years in sprint and endurance distances. Participation data provides evidence that there were equal opportunities to participate in swimming between boys and girls (Fig 3B), thus, we propose that the observed mean sex difference in performance across all freestyle events (8.4%) is *solely* due to physiological differences between the sexes. In support of this, the sex difference in world record swimming performances is similar (8.5%) until ~50 years [13]. Between the ages of 11 and 17 years, the sex difference in performance was strongly associated with circulating testosterone concentrations of boys from a nationally-representative sample (*r* = 0.990, *r*^2^ = 0.980). These data suggest that endogenous testosterone explains 98% of the variance of the sex difference in performance, and support the previous assertion that the sex difference in circulating testosterone of adults explains most of the sex difference in sporting performance [6].

However, prior to the ergogenic effects of puberty/androgen hormones, there are no sex-related differences in performance for the 10^th^-50^th^ places and the top 5 girls have faster performances than the top 5 boys. Importantly, the faster performance of the top girls is clearly not due to earlier initiation of puberty because girls are faster at 5 years of age, well before the age of puberty. Although the precise mechanisms are unclear, these data suggest that girls are inherently faster swimmers than boys (or at least not slower) than boys *sans* the performance-enhancing effects of androgens and puberty. As expected before puberty, there are minimal sex differences between boys and girls in stature [23], hand grip strength [5] and hemoglobin content [24]. Thus, if girls are inherently faster than boys prior to puberty, these sex-based differences would likely be due to optimized composition of the genes encoded on the X chromosome (e.g. genes associated with regulation of blood pressure, angiotensin-related enzymes) in girls with two copies of the X chromosome than boys with only 1 copy of the X chromosome [25]. After puberty however, our data and previous data [5, 6] suggest that ~15× greater concentrations of androgens [21] and subsequent physiological changes in boys compared to girls account for the ~8.5% sex-related difference in performance [6].

These data also demonstrate greater participation in swimming for girls than boys, however, this sex-difference in participation narrows with advancing age (Fig 3B). Limited experimental data exist to explain the greater participation among girls, however, the leading hypothesis is that more girls participate because of longstanding opportunity to participate and compete with (and often outperform) boys. Interestingly, these data also show that despite greater participation for girls than boys, girls have less depth in performance. A prevailing hypothesis (*‘sociocultural conditions hypothesis’* [17]) suggests that decreased opportunities and participation contribute to sex differences in sports performance. Indeed, in a previous study examining collegiate rowing, a sport sanctioned by the US National Collegiate Athletic Association (NCAA) for women but not men, greater participation for women was associated with greater depth of performance for women in the heavyweight class [17]. Thus it is unclear why in this study; girls have greater participation and less depth of performance. However it is clear that these data provide one of the only examples of faster (or at least not slower) sports performance for girls than boys.

## Conclusion

We conclude that prior to the performance-enhancing effects of puberty; the best girls outperform the best boys at sprint and endurance swimming events. Our findings are in direct opposition to nearly universal findings in elite adult athletes that boys are faster than girls. These data provide evidence that the Y chromosome *per se* does not provide an advantage in sports performance. Rather, our data are consistent with ‘doping’ ideology and findings that sustained and augmented levels of endogenous androgens induce performance-enhancing adaptations regardless of genotype of the sex chromosomes. This information may be of use to governing bodies of athletic competitions as eligibility regulations for participation in female events are refined.
